# Quantitative Multilayer Cu(410) Structure and Relaxation Determined by QLEED

**DOI:** 10.1038/s41598-019-52986-w

**Published:** 2019-11-15

**Authors:** Rezwan Ahmed, Takamasa Makino, Jessiel Siaron Gueriba, Seigi Mizuno, Wilson Agerico Diño, Michio Okada

**Affiliations:** 1 Department of Molecular and Material Sciences, Kyushu University, Kasuga, Fukuoka 816-8580 Japan; 2 Department of Chemistry, Osaka University, Toyonaka, Osaka 560-0043 Japan; 3 Department of Applied Physics, Osaka University, Suita, Osaka 565-0871 Japan; 4 Department of Physics, De La Salle University, 2401 Taft Avenue, Manila, 0922 Philippines; 5 Center for Atomic and Molecular Technologies, Osaka University, Suita, Osaka 565-0871 Japan; 6 Institute for Radiation Sciences, Osaka University, Toyonaka, Osaka 560-0043 Japan

**Keywords:** Surfaces, interfaces and thin films, Characterization and analytical techniques, Atomistic models

## Abstract

Industrially relevant catalytically active surfaces exhibit defects. These defects serve as active sites; expose incoming adsorbates to both high and low coordinated surface atoms; determine morphology, reactivity, energetics, and surface relaxation. These, in turn, affect crystal growth, oxidation, catalysis, and corrosion. Systematic experimental analyses of such surface defects pose challenges, esp., when they do not exhibit order. High Miller index surfaces can provide access to these features and information, albeit indirectly. Here, we show that with quantitative low-energy electron diffraction (QLEED) intensity analyses and density functional theory (DFT) calculations, we can visualize the local atomic configuration, the corresponding electron distribution, and local reactivity. The QLEED-determined Cu(410) structure (Pendry reliability factor *R*_P_ ≃ 0.0797) exhibits alternating sequences of expansion (+) and contraction (*−*) (of the first 16 atomic interlayers) relative to the bulk-truncated interlayer spacing of ca. 0.437 Å. The corresponding electron distribution shows smoothening relative to the bulk-determined structure. These results should aid us to further gain an atomic-scale understanding of the nature of defects in materials.

## Introduction

Reactions involve bond-breaking and bond-making^1^. The electrons redistribute (electron dynamics), in an attempt to realize other (meta-) stable configurations. Electron dynamics depend on nuclei/atomic configurations^2^. Nuclei dynamics, in turn, depend on the electron configurations^3^. Eventually, the coupled electronic and atomic dynamics proceed to some final state on the surface^4,5^. Thus, a thorough understanding of the nature of reactions, e.g., catalysis and corrosion, entails an understanding of the elementary dynamical processes involved, in which mass, charge, and energy transport play important roles. Needless to say, this requires knowledge of the (local) atomic configurations. From this we could discern the corresponding electron distribution and local reactivity.

And yet, we still lack a full understanding of the structures of industrially relevant catalytically active surfaces, e.g, copper (Cu)^6–17^. [Cu plays an important role in catalysis, and finds wide utility in applications, e.g., thin film growth, fabrication, and electronics.] This can be attributed to the ubiquitous morphologically rough features, viz., defects in the form of steps and vacancies, esp., if they do not exhibit order. These defects serve as active sites, exposing the incoming adsorbates to both high and low coordinated surface atoms^18–21^. They induce changes in morphology, reactivity, energetics, and/or surface relaxation. These, in turn, affect crystal growth, oxidation, catalysis, and corrosion on the surface^22–29^. Carrying out systematic experimental/structural analyses of such surfaces pose challenges. High Miller index surfaces, which consist of a sequence of terraces (surfaces with low Miller indices) separated by periodic monoatomic steps^30,31^, can provide access to these features and information, albeit indirectly. The surface layers, being exposed to the vacuum, would undergo varying interlayer relaxations, to compensate^32^ for the reduced coordination (as compared to the bulk). The interlayer relaxations would be more prominently observed for high Miller index surfaces, which are more open to the vacuum.

High Miller index Cu surfaces exhibit varied relaxation sequences^6–17,20,22,24–29,31,33^ from which a general rule has been compiled, albeit still contentious^7,31^. Low energy electron diffraction (LEED) analyses report no surface relaxation between the top atomic Cu(410) interlayers^17,30^. On the other hand, ion scattering spectroscopy (ISS) measurements show large contraction between the first few Cu(410) layers^9^. Later investigations suggest that the pattern of relaxation sequences, in broad scale, complement each other. For the case of Ag(410), previous quantitative LEED (QLEED) analyses found no surface relaxation (neither expansion: +nor contraction: −, with respect to the bulk-truncated values) of the first interlayer spacing, and a (−; +) sequence for second and third interlayer spacings, respectively^34^.

Embedded atom models for Ag(410) and Cu(410) show a sequence of contractions for the first, second, and third interlayer spacings, (−; −; −) respectively^10^. All-electron full-potential linearized augmented plane-wave (FLAPW) calculations, in the framework of local density approximation (LDA) and generalized gradient approximation (GGA) studies, of Cu(410) also show a sequence of contractions that continue from the first to the fourth interlayer spacing, i.e., (−; −; −; −)^15^. The discrepancies in the theoretical and experimental results have been attributed to LEED analyses done/limited to only three or four layers from the surface, and do not portray the overall relaxation scenario as depicted by theoretical calculations^15^.

Here, we present results of our experimental study on the Cu(410) surface, using QLEED analyses. To get the optimum (theoretical) energy dependent intensity *I*(*E*) curves, we considered slab thickness ranging from 24 to 64 atomic layers, with corresponding relaxed layers ranging from 8 to 32 atomic layers. We confirmed the consistency of the results with Pendry reliability factors *R*_P_ < 0.2. In the following, we give a detailed discussion of the relaxation (perpendicular and lateral displacements) of the surface atoms.

## Results and Discussions

**Figure 1:**
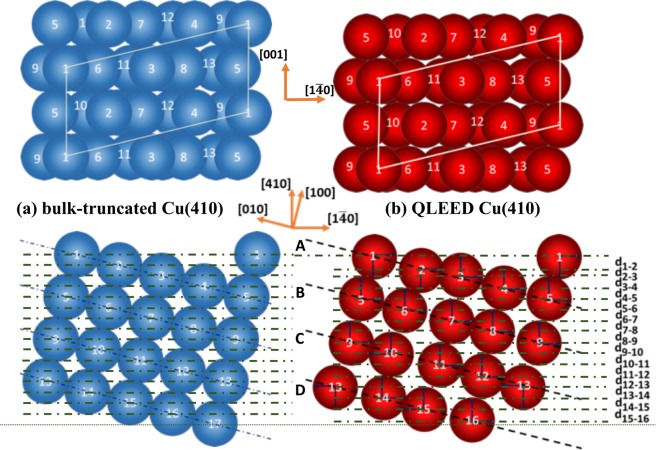
(upper panel) Top and (lower panel) side view of the (**a**) bulk-truncated and (**b**) QLEED-determined relaxed Cu(410). Dotted lines correspond to the positions of the corresponding atomic layers.

**Figure 2:**
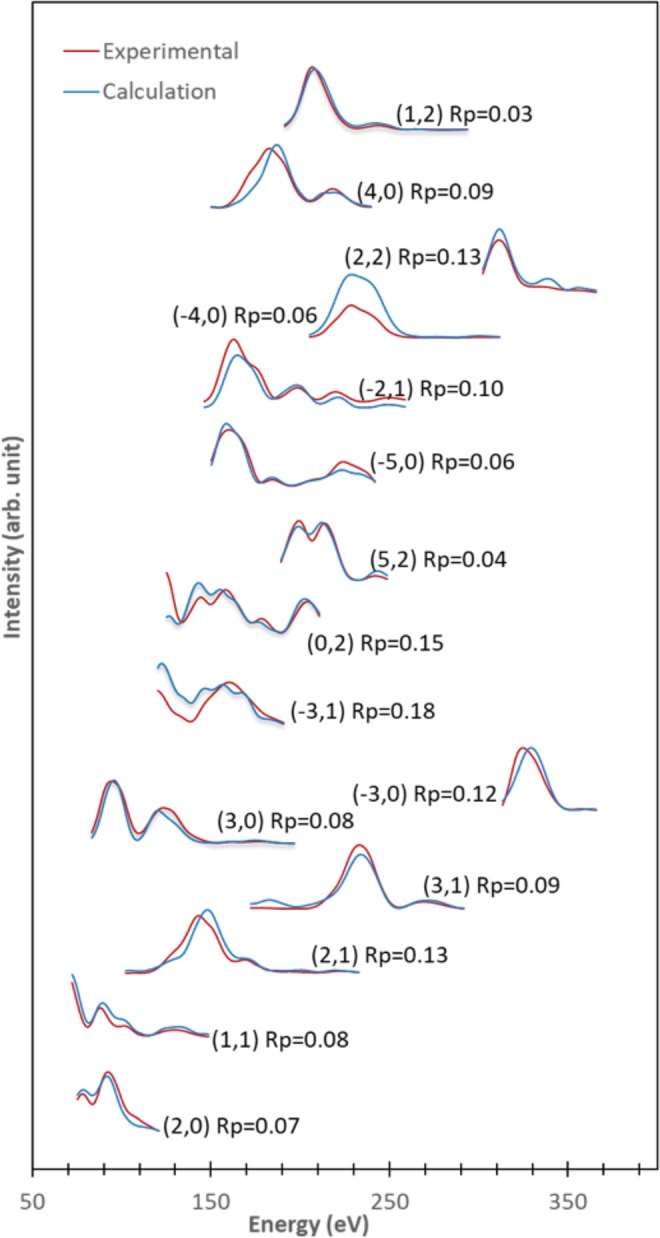
Comparison between the theoretical and experimental *I*(*E*) curves, using a 56 layer thick slab with 16 relaxed layers, with corresponding Pendry reliability factor *R*_P_ = 0.0797.

In Fig. 1, we show a comparison of the interlayer spacing between the bulk-truncated Cu(410) (cf., Fig. 1(a) (lower panel)) and the QLEED-determined relaxed Cu(410) (Fig. 1(b) (lower panel). The theoretical *I*(*E*) curves, determined using 16 optimized relaxed layers, and the experimental *I*(*E*) curves, show good agreement, with *R*_P_ = 0.08 (Fig. 2). We found that Cu(410) exhibits an alternating sequence of expansion (+) and contraction (−), viz., (+; −; +; −; +; −; −), as compared to the bulk-truncated interlayer spacing (ca. 0.437 Å). In Table 1, we show the resulting interlayer relaxation and positions of the top 16 relaxed atoms relative to the bulk crystal.

**Table 1:** Interlayer distance, percentage change ($\Delta {d}_{n,n+1}=\frac{({d}_{n,n+1}-{d}_{bulk})}{{d}_{bulk}}\times 100{\rm{ \% }}$) between the consecutive layers and the bulk, and individual atom positions of the optimized 24.4 Å thick Cu(410) slab (with 56 interlayers, the top 16 layers relaxed, and a corresponding Pendry *R*-factor *R*_P_ = 0.080) relative to the first layer bulk.

Interlayer Distance [Å]						Coordinates [Å]				
	Bulk^b^		Slab^b^			Layer	Bulk^a^		Slab^b^	
	[410]	[1$$\bar{{\bf{4}}}$$0]	[410]	[1$$\bar{{\bf{4}}}$$0]	%[410]		[$$\bar{{\bf{4}}}$$$$\bar{{\bf{1}}}$$0]	[1$$\bar{{\bf{4}}}$$0]	[$$\bar{{\bf{4}}}$$$$\bar{{\bf{1}}}$$0]	[1$$\bar{{\bf{4}}}$$0]
						1	0	0	0.038 ± 0.014	0.173 ± 0.052
*d*_1−2_	0.437	1.754	0.544	1.871	24.577	2	0.437	1.754	0.583 ± 0.016	2.044 ± 0.065
*d*_2−3_	0.437	1.754	0.189	1.557	−56.751	3	0.874	3.508	0.772 ± 0.020	3.601 ± 0.051
*d*_3−4_	0.437	1.754	0.509	1.723	16.430	4	1.311	5.261	1.281 ± 0.012	5.323 ± 0.044
*d*_4−5_	0.437	1.754	0.417	1.787	−4.668	5	1.748	7.015	1.697 ± 0.025	7.110 ± 0.076
*d*_5−6_	0.437	1.751	0.474	1.721	8.535	6	2.185	1.317	2.171 ± 0.024	1.381 ± 0.072
*d*_6−7_	0.437	1.757	0.435	1.864	−0.526	7	2.622	3.073	2.606 ± 0.022	3.245 ± 0.085
*d*_7−8_	0.437	1.752	0.405	1.611	−7.254	8	3.059	4.825	3.011 ± 0.022	4.856 ± 0.066
*d*_8−9_	0.437	1.750	0.459	1.849	5.103	9	3.496	−0.875	3.471 ± 0.023	−0.744 ± 0.053
*d*_9−10_	0.437	1.756	0.451	1.583	3.112	10	3.933	0.881	3.921 ± 0.022	0.839 ± 0.047
*d*_10−11_	0.437	1.752	0.420	1.956	−3.982	11	4.370	2.633	4.341 ± 0.017	2.795 ± 0.066
*d*_11−12_	0.437	1.748	0.441	1.700	0.847	12	4.807	4.381	4.782 ± 0.019	4.494 ± 0.065
*d*_12−13_	0.437	1.765	0.463	1.573	5.927	13	5.244	6.147	5.245 ± 0.022	6.068 ± 0.073
*d*_13−14_	0.437	1.742	0.407	1.920	−6.957	14	5.681	0.439	5.651 ± 0.022	0.538 ± 0.105
*d*_14−15_	0.437	1.758	0.461	1.627	5.538	15	6.118	2.197	6.112 ± 0.023	2.164 ± 0.073
*d*_15−16_	0.437	1.753	0.431	1.898	−1.281	16	6.555	3.95	6.544 ± 0.029	4.062 ± 0.093

Atomic positions and (positive) displacements (towards the bulk, i.e., [$\bar{4}\bar{1}$0]) given in [Å] relative to the topmost atomic layer.

^a^bulk-truncated, unrelaxed

^b^relaxed surface.

### Relaxation of Cu(410)-[100] *vs*. Cu(100)

To validate the results, we compare the interlayer spacings of the terrace atoms of Cu(410), i.e., atoms along Cu(410)-[100] and their Cu(100) counterpart (cf., Fig. 1(b) lower panel and Table 2). To define the first (topmost) interlayer spacing, we took the difference in the average positions of the first group of four atoms, viz., (1, 2, 3, 4) = A layer, and that of the second group of four atoms, viz., (5, 6, 7, 8) = B layer. We did the same for the next two groups of four atoms, viz., C and D layers. The results show A-B contraction, B-C expansion, and no change in C-D (relative to the bulk-truncated (100) interlayer spacing *d*_0_^*bulk*[100]^ = 1.75 Å, cf., Table 2). This trend agrees with the surface layer relaxation of Cu(100)^35^. The topmost and the second layers of the Cu(100) contract relative to the bulk-truncated values, compensating for the lower coordination number. On the average, the Cu(410)-[100] atoms also show similar (A-B) contraction.

**Table 2:** Measured Cu(410) and Cu(100) interlayer distances along [100] show similar relaxation trends relative to the bulk.

[100] layer	Interlayer Distance [Å]			
	Cu(410) bulk-truncated, unrelaxed	Cu(410) measured^a^	Cu(100) bulk-truncated, unrelaxed	Cu(100) measured^b^
A-B	1.75	1.70	1.81	1.77
B-C	1.75	1.76	1.81	1.83
C-D	1.75	1.75	1.81	1.81

^a^This work (QLEED).

^b^From ref. ^9^. (Ion Scattering Spectroscopy).

### Multi-layer relaxation of Cu(410)

As shown in Table 1, the corrugated Cu(410) (cf., Fig. 1) exhibits a complicated relaxation sequence. To smoothen^32^ the electron (density) corrugation, some of the atoms would have to rise upward in the [410] direction. Thus, consistent with the (A-B and C-D) contraction of the [100] atoms, the 16 relaxed surface layers measure 6.518 Å thick (0.037 Å less than the 6.555 Å thick 16 bulk-truncated surface layers, cf., Table 1).

### *Tracking the surface perpendicular and lateral atom displacements*

From Table 1, we see an expansion of the interlayer spacing of the top two atoms along [410] (viz., *d*_1−2_). This results from the positive surface perpendicular and parallel/lateral displacements (along [$$\bar{4}$$$$\bar{1}$$0] and [1$$\bar{4}$$0], respectively) of both Cu atoms 1 and 2 with respect to their corresponding bulk-truncated positions. On the other hand, we see surface perpendicular displacements of both Cu atoms 3 and 4 in the opposite direction ([410]), to reduce surface corrugation^32^. This results in interlayer contraction (cf., *d*_2−3_). With Cu atom 4 also having been displaced slightly along [410] (although with a smaller magnitude due to damping effect), we observe interlayer expansion between Cu atoms 3 and 4 (cf., *d*_3−4_). Cu atom 5, which is partially below Cu atom 1, couples with Cu atom 1, and displaced along [410]. This results in interlayer contraction between Cu atoms 4 and 5 (cf., *d*_4−5_ Table 1), and shortening of the bond length between Cu atoms 1 and 5 to *B*_1−5_ = 2.53 Å. This can be compared to the corresponding bulk-truncated value of 2.55 Å. Cu atoms 6, 7, 8, which are almost at the bottom the four-fold hollow site between the first five top atoms, move upward in the [100] direction relative to their bulk positions. As a result, the interlayer spacings *d*_5−6_, *d*_6−7_ and *d*_7−8_ show the following relaxation sequence, i.e, (+; −; −), respectively. *B*_2−3_ and *B*_7−8_ shows bond-length contraction as compared to their bulk-truncated values, indicating coupling between them. *B*_1−2_, *B*_4−5_, and *B*_6−7_ show bond-length expansion, indicating weakened bonding, as shown in Table 3. The stabilization of the surface atoms and the reduction of the surface free energy near the steps can be attributed to this coupling and relaxation. But, the detailed significance still remains to be understood.

**Table 3:** Bond length between corresponding surface atoms of Cu(410).

Bond lengths [Å]	Bulk	Surface
*B*_1−2_	2.56	2.63
*B*_2−3_	2.56	2.40
*B*_3−4_	2.56	2.59
*B*_4−5_	2.56	2.53
*B*_6−7_	2.56	2.45
*B*_7−8_	2.55	2.63
*B*_1−5_	2.55	2.53

### Cu(410) electron distribution

**Figure 3:**
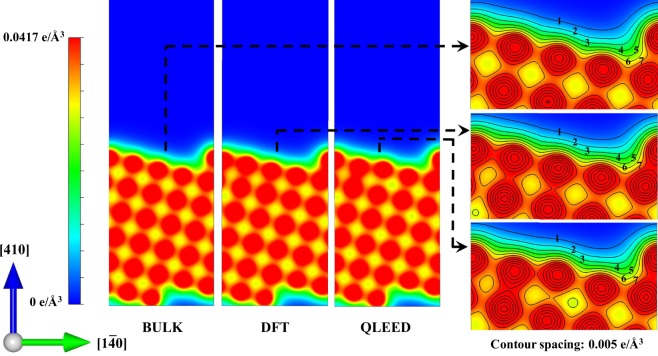
Charge distributions of the BULK-truncated, DFT-optimized, and QLEED-determined structures taken along a cut through a plane along [1$\bar{4}$0]. Color range as shown in the leftmost panel. Corresponding contour plots of the charge distributions are also shown in the rightmost panel. Contour spacing: 0.005 e/Å^3^, with the origin far from Cu(410). Structure drawn using the VESTA package^51^.

Starting from the (accurate) QLEED-determined structure (carried at 120 K), we can determine the corresponding electron distribution of the relaxed Cu(410), within the framework of the density functional theory (DFT)^36,37^. For comparison, we also calculated the corresponding electron distribution for the BULK-truncated structure, and the DFT-optimized (0 K) structure (cf., Fig. 3). Due to the elevation of the terrace atoms, both DFT-optimized and QLEED-determined structures show relative smoothening of the corresponding electron distributions, as compared to the BULK-truncated structure. As in previous theoretical studies^7,31^, the DFT-optimized structure shows a (−; −; −; −; +) relaxation sequence (cf., Table 4). (Apparently, increasing the number of atomic layers considered for the LEED analyses did not resolve the difference with previous theoretical studies, contrary to previous expectations^15^.) It should be noted that the QLEED-determined structure locates at an energy state ca. 0.03 eV higher than that of the bulk-truncated structure. In comparison, the DFT-optimized structure has a corresponding energy ca. 0.07 eV below that of the bulk-truncated structure. The 120 K difference in working temperature, could sustain the QLEED structure, through the excitation of surface phonons/oscillator states (cf., e.g, refs^38,39^ and references therein) and other anharmonic effects, which are not considered in the ground state total energy calculation.

**Table 4:** Calculated interlayer distance [Å] for an optimized 24 layer thick Cu(410) slab, with the top 16 layers relaxed, indicating contraction (−) and/or expansion (+) relative to the bulk.

Interlayer distance [Å]	Bulk^a^	Slab^b^	*Z*-displacement [Å] along [410]	%*Z*-displacement along [410]
*d*_1−2_	0.441	0.391	−0.05	−11.37
*d*_2−3_	0.441	0.405	−0.04	−8.20
*d*_3−4_	0.441	0.369	−0.07	−16.19
*d*_4−5_	0.441	0.426	−0.01	−3.31
*d*_5−6_	0.441	0.499	0.06	13.34
*d*_6−7_	0.441	0.430	−0.01	−2.51
*d*_7−8_	0.441	0.424	−0.02	−3.85
*d*_8−9_	0.441	0.448	0.01	1.60
*d*_9−10_	0.441	0.440	−0.001	−0.22
*d*_10−11_	0.441	0.441	0.001	0.002
*d*_11−12_	0.441	0.454	0.014	3.1
*d*_12−13_	0.441	0.436	−0.004	−1.00
*d*_13−14_	0.441	0.448	0.007	1.59
*d*_14−15_	0.441	0.435	−0.006	−1.36
*d*_15−16_	0.441	0.438	−0.003	−0.66
*d*_16−17_	0.441	0.452	0.011	2.56

^a^bulk truncated, unrelaxed surface.

^b^relaxed surface.

## Conclusions

Here, we showed that with quantitative low-energy electron diffraction (QLEED) intensity analyses and density functional theory (DFT) calculations, we can visualize the local atomic configuration, the corresponding electron distribution, and local reactivity. The QLEED-determined Cu(410) structure exhibits alternating sequences of expansion (+) and contraction (−) (of the first 16 atomic interlayers) relative to the bulk-truncated interlayer spacing of ca. 0.437 Å, with a Pendry reliability factor *R*_P_≃0.0797). The corresponding electron distribution shows smoothening relative to the bulk-determined structure. Thus, we demonstrate how high Miller index surface, viz., Cu(410), can serve as an intermediate stage for analyzing and understanding the role of defects on the atomically flat surface, e.g., how they change their physical and chemical properties, how adsorbates behave near surface defects, and the adsorption mechanism. [Note that the method presented here is not a “fail-proof” approach for surface structure determination. Results from previous studies indicate special care should be taken in determining the surface structures of more complex systems, e.g., oxide surfaces, with reconstructions, and defects (cf., e.g., refs^40–42^)]. Further detailed structural study of the Cu(410) and the corresponding local reactivity^43,44^ can be done by adsorbing hydrogen, oxygen or silicon, and LEED analyses. This detailed interlayer relaxation (including the perpendicular and lateral displacements of the surface atoms) of high Miller index Cu(410) may also yield a better understanding of the merits and limitations of widely used experimental and theoretical methods for surface structural analyses.

## Methods

### Quantitative low energy electron diffraction analyses

We performed the experiments in an ultrahigh-vacuum (UHV) chamber equipped with a four grid LEED system, with the base pressure in the chamber kept at 5 × 10^−8^ Pa. We cleaned the Cu(410) sample by repeated cycles of Ar^+^ sputtering (1 kV, 7*μ*A, 15 min), followed by subsequent annealing, until we obtain a sharp LEED pattern. After cooling the crystal to 120 K using liquid N_2_, we recorded the LEED pattern for the clean Cu(410) using a digital charge-coupled device (CCD) camera with a computer-controlled data acquisition (DAQ) system. In Fig. S1 we show the Cu(410) LEED pattern at 120 eV and 220 eV, with the single mirror plane and the unit cell of the reciprocal lattice indicated. To calculate the change in intensity for the theoretical *I*(*E*) curves between the range 70 to 400 eV, we use the Barbieri/Van Hove symmetrized automated tensor LEED package. We fixed the imaginary part of the inner potential *V*_0*i*_ = −5.0 eV, and determined the real part using theoretical and experimental best fits. To obtain the best fit model (via agreement between the experimental and theoretical *I*(*E*) curves), we recorded 15 symmetrically inequivalent beams, for a total energy range of 5285 eV. To calculate the theoretical *I*(*E*) curves, we need to consider a surface thick enough to diffract all the incident electrons in the energy range 70–400 eV. The procedure is not as straightforward as choosing the top 8 atomic layers of the Cu(410) unit cell (cf., Fig. 1a). The layer spacing between neighboring bulk-truncated Cu atoms *d*_0_^*bulk*[410]^ = 0.437 Å in [410] and *d*_0_^*bulk*[1^${}^{\bar{4}}$^0]^ = 1.754 Å in [1$\bar{4}$0] (considering a Cu lattice constant *a*_C*u*_ = 3.615 Å), respectively. The top 8 atomic layers (which corresponds to a thickness of ca. 3.50 Å, assuming *d*_0_ = 0.437 Å) would not be thick enough. Furthermore, these atoms will relax along the [410] and the [1$\bar{4}$0] directions, to smoothen the electron charge density^32^. (*pm* symmetry restricts movement in the [001] direction.) So, to determine the appropriate surface thickness, we considered increasing number of atomic layers until the corresponding Pendry reliability factor *R*_P_ converges to a low value with consistent interlayer relaxations. We found that, until 32 atomic layers, the interlayer displacements and the corresponding *R*_P_ values vary with (increasing) number of atomic layers, beyond which they converge. Finally, we chose to consider a 56 layer, ca. 24.4 Å thick, slab for further analyses. (Results for 24, 32, 40, 48, 56, 64 atomic layer thick surfaces considered can be found in Supplementary Table S1). For renormalized forward scattering approximation, we determined that the layer spacing should be at least 0.9 Å. Between the surface and the bulk layer, we put a 0.9 Å spacing throughout the calculation. The choice of a sufficiently thick surface layer ensures that this spacing has no effect on our results. The error bars for the structural parameters are calculated from the variance of *R*_P_, i.e., Δ*R* = *R*_*min*_(8|*V*_0*i*_|/Δ*E*)^1/2^, where *R*_*min*_ = 0.0797 (*R*_*min*_: minimum *R*_P_) and Δ*E* = 5285 eV (Δ*E*: total energy range of the experiment). In addition, if we consider the relaxation of a surface unit cell consisting 8 Cu atoms, then a total of 16 structural parameters influence optimization. As we increase the number of layers responsible for relaxation, the number of structural parameters also increase as atoms in deeper layers would also influence surface Cu atom relaxations. However, increasing the number of relaxed parameters makes it difficult for the optimized structure to reach the global minimum. Therefore, we gradually increased the number of layers, considering 4, 8, 16, 24, and 32 relaxed layers, searching for the global minimum, while keeping the slab thickness fixed at 56 layers. [Note that the unit cell of a clean Cu(410) corresponds to at least 8 layers, so optimizing less than 8 layers may not portray the true picture of the relaxation, and could even give misleading data.] The decrease in the corresponding Pendry reliability factor values with increasing number of relaxed layers (i.e., *R*_P_ = 0.096, 0.093, 0.078, 0.066, 0.061, respectively) indicate improved agreement in the theoretical-experimental *I*(*E*) curves. And they all give similar relaxation sequence for the topmost layers. We also see that the *R*_P_ values begin to converge from 24 layers onwards. So, for our final analyses, we considered optimizing 16 layers (ca. 6.992 Å), while keeping the slab thickness fixed at 56 layers. In a previous study using LEED^35^, we were able to determine the structure of the first four top layers of clean Cu(001), which is ca. 7 Å thick. To account for thermal vibrations, we used a Debye temperature of 230 K, optimized for the top 8 layers of the unit cell (as in previous studies for clean Cu(001)^35^). Also note that the corresponding relaxations and Pendry reliability factors remain constant for the optimized surface Debye temperature range 175 K to 300 K, while keeping the bulk Debye temperature constant at 343 K.

### Density functional theory (DFT)-based total energy calculations; surface structure optimization

We performed density functional theory (DFT)-based total energy calculations^36,37^, using projector augmented wave (PAW) formalism^45^, with Perdew-Burke-Enzerhoff (PBE) generalized gradient (GGA) exchange correlation functional^46–49^, and a cutoff energy of 550 eV. We adopt the Monkhorst and Pack method^50^ to perform the Brillouin zone integrations, with 9 × 9 × 1 special *k*-points. The optimized structures of the bulk and surfaces are obtained with an energy convergence of less than 10^−5^ eV and that the Hellman-Feynman forces acting on each atom be below 0.01 eV/Å. The calculated optimized bulk lattice parameters for Cu* a*_C*u*_ = 3.634 Å. To model Cu(410), we used a periodic slab 24 Cu atomic layers thick ((1 × 1) surface unit cell, with one Cu atom per layer, topmost 16 layers allowed to relax and the last 8 layers held at their bulk-truncated positions), separated by 15 Å of vacuum along [410].

## Supplementary information

Supplementary Information
