# Supplementary material for: Quantitative Multilayer Cu(410) Structure and Relaxation Determined by QLEED
Source: Sci Rep. 2019 Nov 15;9:16882. doi: 10.1038/s41598-019-52986-w (PMC6858363; doi:10.1038/s41598-019-52986-w)
Supplement: Supplementary file 1 — Supplementary Information [file 41598_2019_52986_MOESM1_ESM.pdf]

Supplementary Information

Quantitative Multilayer Cu(410) Structure and Relaxation Determined by QLEED

Rezwan Ahmed[1], Takamasa Makino[2], Jessiel Siaron Gueriba[3,4],
Seigi Mizuno[1,*], Wilson Agerico Diño[3,5,**], and Michio Okada[2,6,***]
[1]Department of Molecular and Material Sciences, Kyushu University, Kasuga, Fukuoka 816-8580, Japan
[2]Department of Chemistry, Osaka University, Toyonaka, Osaka 560-0043, Japan
[3]Department of Applied Physics, Osaka University, Suita, Osaka 565-0871, Japan
[4]Department of Physics, De La Salle University, 2401 Taft Avenue, Manila 0922, Philippines
[5]Center for Atomic and Molecular Technologies, Osaka University, Suita, Osaka 565-0871, Japan
[6]Institute for Radiation Sciences, Osaka University, Toyonaka, Osaka 560-0043, Japan
* mizuno.seigi@kyudai.jp
** wilson@dyn.ap.eng.osaka-u.ac.jp
*** okada@chem.sci.osaka-u.ac.jp

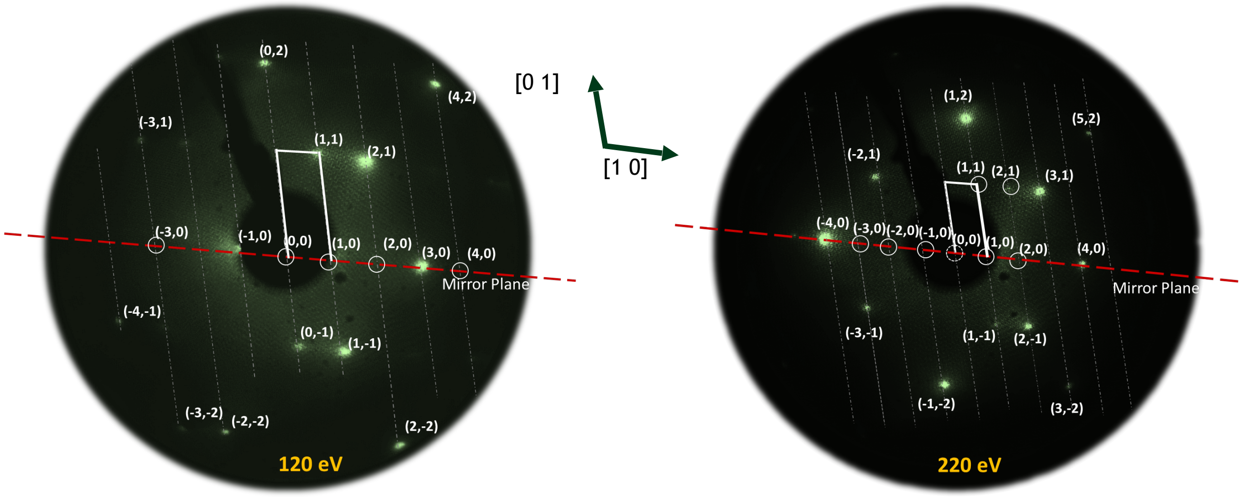

Figure S1. LEED pattern of a clean Cu(410) taken at (left panel) 120 eV and (right panel) 220 eV beam energies. Red and white lines indicate the *pm* mirror plane and the unit cell of the reciprocal lattice, respectively. The white circles represent missing spots, which appear over the course of changing beam energies.

Table S1. Structure parameters for Cu(410), determined by varying the slab thickness, with the number of relaxed layers fixed at 16 layers.

			# of relaxed layers	16		# of relaxed layers	16		# of relaxed layers	16		# of relaxed layers	16		# of relaxed layers	16		# of relaxed layers	16	
	Bulk		# of slab layers	24		# of slab layers	32		# of slab layers	40		# of slab layers	48		# of slab layers	56		# of slab layers	64	
Interlayer Distance [Å]	[410]	[1$\bar{4}$0]	[410]	[1$\bar{4}$0]	%[410]	[410]	[1$\bar{4}$0]	%[410]	[410]	[1$\bar{4}$0]	%[410]	[410]	[1$\bar{4}$0]	%[410]	[410]	[1$\bar{4}$0]	%[410]	[410]	[1$\bar{4}$0]	%[410]
d_{1-2}	0.437	1.7538	0.5454	1.8711	24.8055	0.5468	1.8745	25.1259	0.5459	1.8789	24.9199	0.5484	1.8401	25.492	0.5444	1.8709	24.5767	0.5431	1.8754	24.2792
d_{2-3}	0.437	1.7538	0.2095	1.5648	-52.0595	0.188	1.5672	-56.9794	0.1884	1.5647	-56.8879	0.1902	1.6005	-56.476	0.189	1.5569	-56.7506	0.1925	1.5708	-55.9497
d_{3-4}	0.437	1.7538	0.5085	1.7295	16.3616	0.5057	1.7075	15.7208	0.5079	1.7166	16.2243	0.5069	1.7226	15.9954	0.5088	1.7228	16.4302	0.5079	1.6951	16.2243
d_{4-5}	0.437	1.7538	0.4283	1.7867	-1.9908	0.4198	1.7848	-3.9359	0.4165	1.7815	-4.6911	0.4156	1.778	-4.897	0.4166	1.7865	-4.6682	0.4118	1.802	-5.7666
d_{5-6}	0.437	1.7510	0.4791	1.7649	9.6339	0.4782	1.7517	9.4279	0.4756	1.7373	8.833	0.4767	1.7363	9.0847	0.4743	1.7212	8.5355	0.4841	1.7341	10.778
d_{6-7}	0.437	1.7568	0.4033	1.7964	-7.7117	0.4275	1.8263	-2.1739	0.4291	1.8436	-1.8078	0.4329	1.8435	-0.9382	0.4347	1.8641	-0.5263	0.4199	1.8373	-3.913
d_{7-8}	0.437	1.7517	0.4448	1.641	1.7849	0.415	1.6319	-5.0343	0.4136	1.6299	-5.3547	0.3948	1.6308	-9.6568	0.4053	1.611	-7.254	0.4244	1.6379	-2.8833
Coordinates																				
1	0	0	-0.0002	0.1662		0.0382	0.1662		0.0402	0.1584		0.0365	0.1588		0.0383	0.1727		0.037	0.1676	
2	0.437	1.7538	0.5452	2.0373		0.585	2.0407		0.5861	2.0373		0.5849	1.9989		0.5827	2.0436		0.5801	2.043	
3	0.874	3.5076	0.7547	3.6021		0.773	3.6079		0.7745	3.602		0.7751	3.5994		0.7717	3.6005		0.7726	3.6138	
4	1.311	5.2614	1.2632	5.3316		1.2787	5.3154		1.2824	5.3186		1.282	5.322		1.2805	5.3233		1.2805	5.3089	
5	1.748	7.0152	1.6915	7.1183		1.6985	7.1002		1.6989	7.1001		1.6976	7.1		1.6971	7.1098		1.6923	7.1109	
6	2.185	1.3165	2.1706	1.4335		2.1767	1.4022		2.1745	1.3877		2.1743	1.3866		2.1714	1.3813		2.1764	1.3953	
7	2.622	3.0733	2.5739	3.2299		2.6042	3.2285		2.6036	3.2313		2.6072	3.2301		2.6061	3.2454		2.5963	3.2326	
8	3.059	4.825	3.0187	4.8709		3.0192	4.8604		3.0172	4.8612		3.002	4.8609		3.0114	4.8564		3.0207	4.8705	

Table S2. Structure parameters for Cu(410), determined by varying the number of relaxed top atomic layers, with slab thickness fixed at 56 layers, and the corresponding Pendry reliability factors R_P.

			# of relaxed layers: 8		R_P	# of relaxed layers: 16		R_P	# of relaxed layers: 24		R_P
	Bulk		# of slab layers: 56		0.07	# of slab layers: 56		0.078	# of slab layers: 56		0.06
Interlayer Distance [Å]	[410]	[$1\bar{4}0$]	[410]	[$1\bar{4}0$]	%[410]	[410]	[$1\bar{4}0$]	%[410]	[410]	[$1\bar{4}0$]	%[410]
d_{1-2}	0.437	1.7538	0.5372	1.8833	22.9291	0.5444	1.8709	24.5767	0.5532	1.8462	26.59039
d_{2-3}	0.437	1.7538	0.2038	1.5964	-53.3638	0.189	1.5569	-56.7506	0.182	1.5997	-58.3524
d_{3-4}	0.437	1.7538	0.5078	1.6971	16.2014	0.5088	1.7228	16.4302	0.5018	1.7036	14.82838
d_{4-5}	0.437	1.7538	0.4153	1.7914	-4.9657	0.4166	1.7865	-4.6682	0.4312	1.7802	-1.32723
d_{5-6}	0.437	1.7510	0.4748	1.7528	8.6499	0.4743	1.7212	8.5355	0.4628	1.7390	5.90389
d_{6-7}	0.437	1.7568	0.4368	1.8688	-0.0458	0.4347	1.8641	-0.5263	0.4394	1.871	0.549199
d_{7-8}	0.437	1.7517	0.3980	1.5852	-8.9245	0.4053	1.611	-7.254	0.3987	1.5982	-8.7643
Coordinates											
1	0	0	0.0261	0.0977		0.0383	0.1727		0.0298	0.1702	
2	0.437	1.7538	0.5633	1.981		0.5827	2.0436		0.583	2.0164	
3	0.874	3.5076	0.7671	3.5774		0.7717	3.6005		0.765	3.6161	
4	1.311	5.2614	1.2749	5.2745		1.2805	5.3233		1.2668	5.3197	
5	1.748	7.0152	1.6902	7.0659		1.6971	7.1098		1.698	7.0999	
6	2.185	1.3165	2.165	1.369		2.1714	1.3813		2.1608	1.3892	
7	2.622	3.0733	2.6018	3.2378		2.6061	3.2454		2.6002	3.2602	
8	3.059	4.825	2.9998	4.823		3.0114	4.8564		2.9989	4.8584	

Table S3. Displacements of the optimized surface atoms relative to its bulk-state coordinates. Positive displacements along the [410] (towards vacuum) and negative displacements along $[\bar{4}\bar{1}0]$ (in the opposite direction towards the bulk).

DFT		QLEED	
Bulk z-coordinate	Slab displacement [Å]	Bulk z-coordinate	Slab displacement [Å]
0	-0.117	0	-0.038
-0.441	-0.067	-0.437	-0.146
-0.881	-0.03	-0.874	0.102
-1.322	0.041	-1.311	0.03
-1.763	0.055	-1.748	0.051
-2.203	-0.003	-2.185	0.014
-2.644	0.008	-2.622	0.016
-3.084	0.025	-3.059	0.048
-3.525	0.018	-3.496	0.025
-3.966	0.019	-3.933	0.012
-4.406	0.019	-4.37	0.029
-4.847	0.005	-4.807	0.025
-5.288	0.009	-5.244	-0.001
-5.728	0.002	-5.681	0.03
-6.169	0.008	-6.118	0.006
-6.61	0.011	-6.555	0.011
